# ATP Enhances Spontaneous Calcium Activity in Cultured Suburothelial Myofibroblasts of the Human Bladder

**DOI:** 10.1371/journal.pone.0025769

**Published:** 2011-10-05

**Authors:** Sheng Cheng, Frank Peter Scigalla, Pietro Speroni di Fenizio, Zhi Geng Zhang, Jens-Uwe Stolzenburg, Jochen Neuhaus

**Affiliations:** 1 Department of Urology, University of Leipzig, Leipzig, Germany; 2 Department of Urology, Sir Run Run Shaw Hospital, College of Medicine, Zhejiang University, Hangzhou, China; 3 Department of Informatics Engineering, CISUC, University of Coimbra, Coimbra, Portugal; University of California Merced, United States of America

## Abstract

**Background:**

Suburothelial myofibroblasts (sMF) are located underneath the urothelium in close proximity to afferent nerves. They express purinergic receptors and show calcium transients in response to ATP. Therefore they are supposed to be involved in afferent signaling of the bladder fullness. Since ATP concentration is likely to be very low during the initial filling phase, we hypothesized that sMF Ca^2+^ activity is affected even at very low ATP concentrations. We investigated ATP induced modulation of spontaneous activity, intracellular calcium response and purinergic signaling in cultured sMF.

**Methodology/Principal Findings:**

Myofibroblast cultures, established from cystectomies, were challenged by exogenous ATP in presence or absence of purinergic antagonist. Fura-2 calcium imaging was used to monitor ATP (10^−16^ to 10^−4^ mol/l) induced alterations of calcium activity. Purinergic receptors (P2X1, P2X2, P2X3) were analysed by confocal immunofluorescence. We found spontaneous calcium activity in 55.18%±1.65 of the sMF (N = 48 experiments). ATP significantly increased calcium activity even at 10^−16^ mol/l. The calcium transients were partially attenuated by subtype selective antagonist (TNP-ATP, 1 µM; A-317491, 1 µM), and were mimicked by the P2X1, P2X3 selective agonist α,β-methylene ATP. The expression of purinergic receptor subtypes in sMF was confirmed by immunofluorescence.

**Conclusions/Significance:**

Our experiments demonstrate for the first time that ATP can modulate spontaneous activity and induce intracellular Ca^2+^ response in cultured sMF at very low concentrations, most likely involving P2X receptors. These findings support the notion that sMF are able to register bladder fullness very sensitively, which predestines them for the modulation of the afferent bladder signaling in normal and pathological conditions.

## Introduction

The perception of bladder filling is essential for the control of bladder function. Most of the storage urinary symptoms, such as urinary urgency, increased frequency of micturition and urgency incontinence can be explained by pathologically enhanced bladder fullness sensation.

The urothelium releases a number of signaling molecules onto stretch activation during the filling phase of the bladder. ATP, an important neurotransmitter, is released from the urothelium during bladder distension [1], [2]. This is presumably the first step in the excitation of bladder afferents as the bladder fills with urine. This hypothesis was strengthened by the localization of P2X3 receptors on suburothelial nerves [3] and the fact that the micturition reflex was reduced in P2X3 knockout mice [4]. The bladder also elicits spontaneous transient rises in intravesical pressure during the filling phase prior to the micturition in intact bladder [5], [6]. The details of the mechanisms and the cell types involved in spontaneous activity are unclear.

Recently, myofibroblastic cells have been identified in the lamina propria of the human and other species [7]–[9]. Those cells form a distinct layer underneath the urothelium in close proximity to afferent nerves [8] and we therefore refer to these cells as suburothelial myofibroblasts (sMF). There is an ongoing debate as to whether these cells are indeed interstitial cells of Cajal (ICCs) as promoted by McCloskey in a recent review [10]. However, while c-kit positive cells resembling ICCs are numerous in guinea-pig and pig bladders only a subpopulation of vimentin (vim) and alpha-smooth muscle cell actin (aSMCA) positive cells also stain positive for c-kit [7], [11]. Suburothelial aSMCA^+^/vim^+^ positive cells of typical irregular blistered shape, which are clearly different from smooth muscle cells, are most frequent in the lamina propria of the human bladder [12].

Suburothelial myofibroblasts, which are characterized by the expression of gap-junction protein Cx43 and the formation of functional syncytia [11], [12], show spontaneous Ca^2+^ activity [9], [13] and are able to generate intracellular Ca^2+^ transients in response to exogenous ATP application [14]; several purinergic receptors have been observed in sMF [9], [15]. The location of sMF and their responsiveness to ATP place them in an ideal position to act as modulators of sensory processes.

Since the physiological ATP concentration during the initial filling phase is likely to be very low, we hypothesized that the Ca^2+^ activity of the sMF is affected at very low ATP concentrations. Furthermore, it was hypothesized that the spontaneous activity of the sMF is likely to be connected with the generation or amplification of the afferent signals [8], [16]. Thus the autonomous activity of the detrusor could be 'triggered' by sMF activity.

In the present study we investigated the ATP induced modulation of spontaneous activity, intracellular calcium response, and purinergic signaling in cultured human suburothelial myofibroblasts.

## Materials and Methods

### Ethics Statement

The study was approved by the Ethics Committee of the University of Leipzig (#036—2007) and was conducted according to the principles expressed in the Declaration of Helsinki. Written informed consent was obtained from all patients.

### Cell cultures

We used tumor free bladder tissue samples from patients undergoing radical cystectomy due to bladder cancer. For setup of hsMF we separated mucosa and lamina propria from muscularis by sharp dissection, ensuring no contamination with detrusor smooth muscle cells. Thus aSMCA and vimentin positive cells were the most abundant cell population besides urothelial cells in this part of the bladder (Fig. S1A,B). CD117 positive interstitial cells of Cajal (ICC) were only sparse in human bladder lamina propria and therefore cannot account for the majority of the cultured suburothelial myofibroblasts (Fig. S1C,D,E). Small tissue fragments (0.5×0.5×0.5 mm) were plated into tissue culture flasks (TPP AG, Trasadingen, Switzerland) and incubated at 37°C and 5% CO_2_ in SMC Growth Medium 2 (PromoCell GmbH, Heidelberg, Germany) and subcultured up to the third passage (P3). The growing cells showed typical morphological and immunohistochemical features of myofibroblasts as recently described [17]. The use of special smooth muscle cell growth medium was sufficient to avoid growth of urothelial cells as proven by visual phase contrast (Fig. S2A,B) and lack of cells staining positive for cytokeratin (monoclonal mouse anti-pan cytokeratin, Sigma-Aldrich, Steinheim, Germany; data not shown). For calcium imaging experiments cells were plated onto 13mm glass coverslips coated with collagen A (Biochrome AG, Berlin, Germany) and grown to a confluence of about 80% for the calcium imaging experiments.

### Solutions and chemicals

We used the modified Krebs Ringer solution of the following composition (mM): CaCl_2_, 1.9; NaCl, 120.9; NaHCO_3_, 14.4; KCl, 5.9; MgCl_2_,1.2; NaH_2_PO_4_, 1.55; Hepes, 4.2; Glucose, 11.49; pH 7.2. All solutions were prepared fresh on the day of use. The following chemicals were obtained from Sigma-Aldrich: ATP,α,β-methylene ATP (AMBA), A-317491, DMSO; Fura-2AM and Pluronic® (Invitrogen, Karlsruhe, Germany); TNP-ATP (Torcris Biosciences, Bristol, UK). All chemicals were diluted from in Krebs Ringer solution 10 mM stock solutions stored at −20°C on the day of experiments. ATP and TNP-ATP were kept in the dark. All drugs were diluted in pure Krebs Ringer solution, which was also used for control in the different experimental parts.

### Intracellular calcium measurements

After a brief wash in ringer the coverslips were bulk-loaded with Fura-2-acetoxymethylester (2.5 µM Fura-2AM solved in DMSO, 2% Pluronic in ringer; 22°C; 40 min.). After 15 minutes wash in ringer to ensure cleavage of the acetoxymethyl ester by endogeneous esterases, the cells were superfused for another 10 minutes with carbogenized ringer (pH 7.2) at a constant flow rate of 0.8 ml/min at 37°C in the recording chamber (WPI, RC-26G, 234 µl volume) before calcium measurements.

Calcium imaging recordings were carried out with a cooled TILL IMAGO-QE camera, connected to an Olympus IX-71 inverse microscope. TILLvisION 4.5 (TillPhotonics GmbH, Gräfelfing, Germany) was used for camera control and data acquisition. Image series were recorded every second for 15 minutes with an excitation wavelength of 340 nm and 380 nm. Fura-2 fluorescence ratios were calculated as FI = F_340 nm_/F_380 nm_*1000 after dynamic background subtraction.

### Data analysis with automated Fluorescence analysis

**Figure 1:**
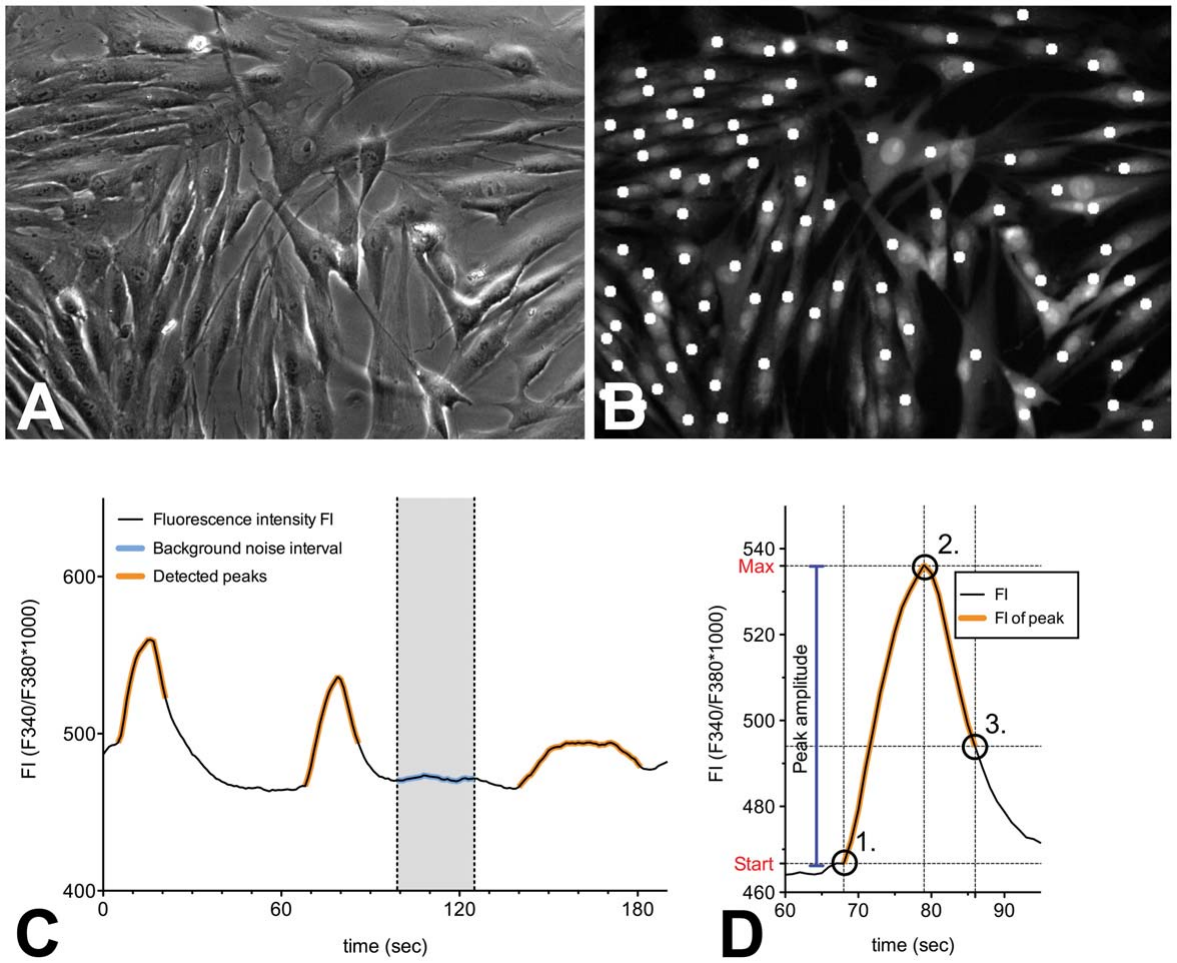
Data analysis with automated fluorescence analysis. (**A**) Phase contrast image of cultured human suburothelial myofibroblasts at about 80% confluency. (**B**) Fura-2 fluorescence image at 380 nm excitation. One circular region of interest (ROI) (white spots) with a diameter of 8.25 µm was defined for each myofibroblast close to the nucleus. (**C**) Automated fluorescence analysis for calcium kinetics. Black line: filtered fluorescence ratio (F340/380); Blue: interval of background noise; Peak starts were defined, when the signal-increase exeeded 2.5x the standard deviation of DF340/380 ( = F340/380 - background). (**D**) Computation of the peak intervalls. Orange: Detected peak intervals; the program tracked the global maximum (2) after a peak start (1). The end of the peak (3) was determined as decline of the signal strength below 60% of the signal amplitude.

We used a self written ImageJ-script (Rasband WS (1997–2006), NIH USA, http://rsb.info.nih.gov/ij/) to calculate the fluorescence intensity (FI) kinetics of the region of interest (ROIs) (Fig. 1A, B). The following analysis of the calcium kinetics was performed with a self programmed automated fluorescence analysis (FA), which is a self-written script for Python 2.6 (Rossum G (2008) Python/C, Python Software Foundation, http://python.org/). The FA was used to detect and characterize the peaks within the timecourse of the Ca^2+^ fluorescence. After detection of the signals, the FA program identifies signal characteristics for the whole observation interval and for each detected peak separately, including peak amplitude and number of peaks (Fig. 1C, D).

**Figure 2:**
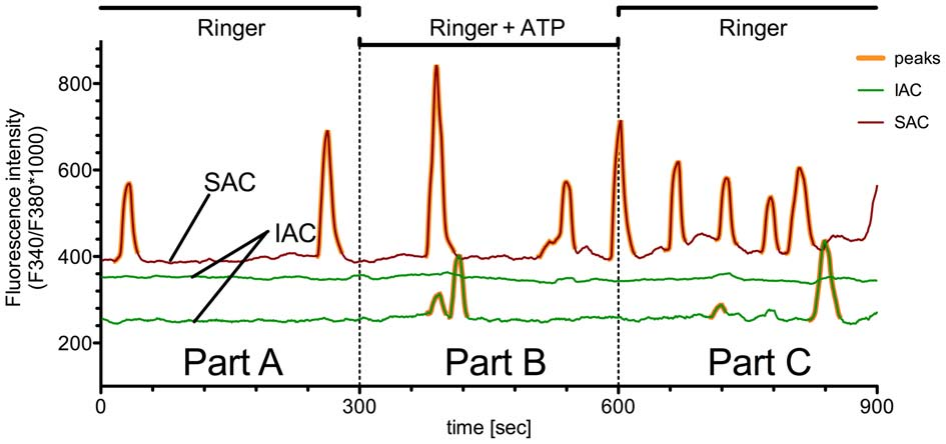
Calcium activity characteristics of cultured human suburothelial myofibroblasts (sMF). Continuous recording of calcium fluorescence intensity. Cells were overflown with ringer or vehicle control in Part A and Part C and exposed to agonists in Part B. The representative traces show two examples of calcium activity characteristics of sMF. Spontaneously active cells (SAC) showed initial spontaneous Ca^2+^ transients in Part A. Inactive cells (IAC) showed no initial spontaneous Ca^2+^ transients in Part A.

The experiments consisted of three parts of 5 minutes each (Fig. 2 Part A, B, C). The suburothelial myofibroblasts were superfused with Ringer solution in Part A and Part C in order to assess the spontaneous activity. Cells were defined as ‘spontaneously active cells’ (SAC) if they showed spontaneous calcium transients in Part A of the experimental procedure. In contrast, inactive cells were denoted IAC.

### Confocal Immunofluorescence

Cells were fixed in 4% paraformaldehyde (1 h, 4°C), washed three times in Tris-buffered saline (TBS; pH 7.2), permeabilized for 10 min with DT-TBS (0.08% Triton-X100 and 0.32% dimethyl sulfoxide), and blocked with 3% skimmed milk and 1% bovine serum albumin in TBS. Double labelling experiments were done with rabbit polyclonal anti-P2X1, P2X2, P2X3 (1∶1000, immunoglobulin G, IgG, Sigma-Aldrich), and mouse monoclonal anti-aSMCA (1∶2000, IgG2a; Sigma-Aldrich) antibodies. Alexa-fluor 488 (A-488, Goat-anti-Mouse IgG 2a) and A-555 (Goat-anti-Rabbit) coupled secondary antibodies (Invitrogen) diluted 1∶500 in TBS (1 h, room temperature) were used for visualisation. Nuclei were stained with 4′,6-diamidino-2-phenylindole-dihydrochloride (DAPI; Roche Diagnostics, Mannheim, Germany). Immunolabelled cells were examined with a laser-scanning microscope (LSM5 Pascal; Zeiss, Jena, Germany). The purinergic receptors expression was analysed with ImageJ and MS-Excel (Microsoft, Redmond, USA).

### Statistics

GraphPad Prism (GraphPad 5.0 Software, Inc., San Diego, USA) was used for presentation and statistical analysis of the data by Student's t-test for paired or unpaired data or one-way ANOVA with Tukey's multiple comparison test. A P-value<0.05 was regarded as statistically significant.

## Results

### Spontaneous calcium activity of sMF

We found basic spontaneous calcium activity (Fig. 2, Part A) in 55.18%±1.65 of the sMF, with a mean amplitude of ΔFI = 243.9±10.24 F_340 nm_/F_380 nm_*1000 and an average Ca^2+^ peak frequency of 0.41±0.01 min^−1^ (n = 4039; N = 48). After Part A, we switched the Ringer's solution to a second solution which additionally contained the substances (ATP, Antagonists) and recorded the reaction of the suburothelial myofibroblasts (Fig. 2, Part B).

### ATP effects on calcium response in sMF

**Figure 3:**
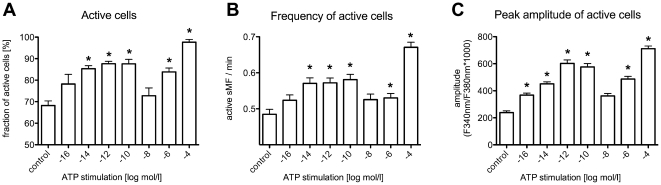
ATP effects on calcium response in sMF. (**A**) fraction of active cells; (**B**) frequency of active cells; (**C**) mean amplitude of active cells. Data are expressed as mean (SEM; N = 6 separate experiments). Note the bi-phasic dose-response curves. *p<0.05 was considered significant comparing ATP-stimulated cells to control (unpaired t-test).

The fraction of active cells, the frequency of active cells and Ca^2+^ peak amplitude increased with increasing ATP concentrations. However, the ATP dose-response curve showed a bi-phasic course with an increase from 10^−16^ to 10^−10^ mol/l and from 10^−6^ to 10^−4^ mol/l and a drop to control level at 10^−8^ mol/l (Fig. 3A, B, C).

**Figure 4:**
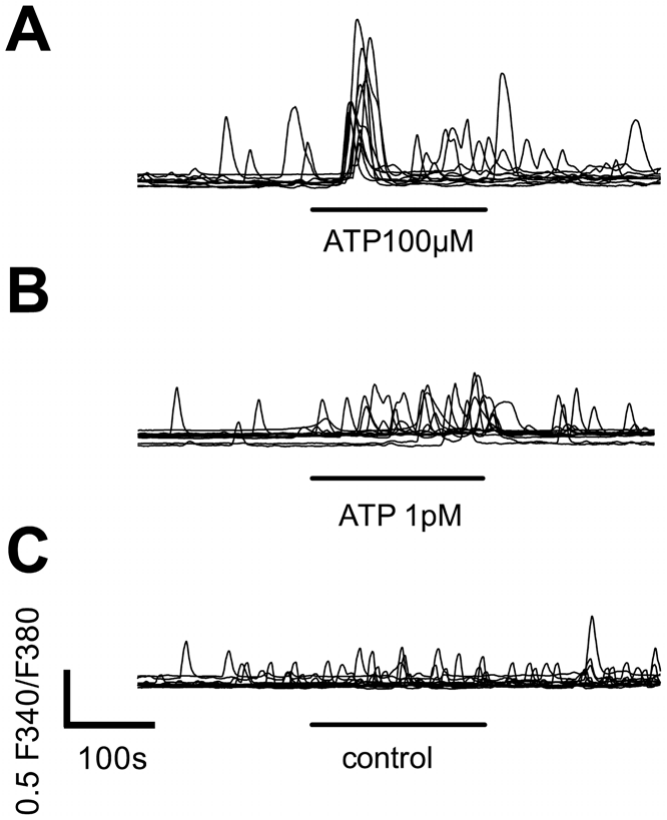
Representative traces of intracellular calcium responses at low and high ATP concentrations. (**A**) The initial peaks emerged immediately after 100 µM ATP stimulation. (**B**) Cells did not show synchronized peaks when challenged with 1pM ATP. However, increased numbers of small calcium transients occurred during ATP stimulation. (**C**) In control experiments (superflow of ringer solution without ATP) almost no significant increase of peaks or peak amplitudes were recorded.

The kinetics of the calcium transients in sMF were different at low and high ATP concentrations. Figure 4 shows representative traces of sMF (control, 10^−12^ and 10^−4^ mol/l ATP). The stimulation with a high ATP concentrations (>10^−8^ mol/l) typically evoked synchronous calcium transients with a rapid initial steep calcium rise (Fig. 4A). The response to the low ATP-concentration of 10^−12^ mol/l did not show the characteristic high fist peak, which we observed at 10^−4^ mol/l ATP. Instead, the first calcium rise varied considerably from cell to cell (Fig. 4B). However, the overall profile was similar to the cells in the control experiments (Fig. 4C), except for higher peak frequency and amplitude.

**Figure 5:**
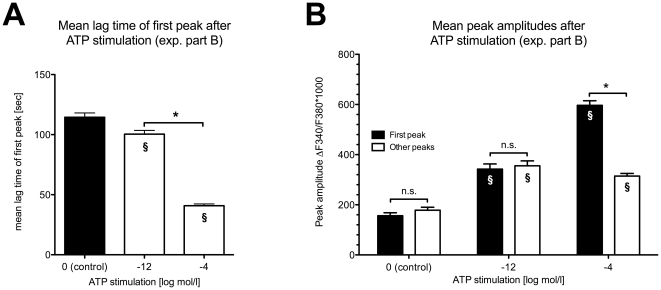
ATP effects on spontaneous activity at low (10^−12^ mol/l, 1pM) and high (10^−4^ mol/l, 100 µM) concentrations. (**A**) The mean lag time of the first peak after ATP stimulation was shorter at 100 µM ATP application than at 1pM ATP application. (**B**) Mean amplitude of the first peak (black columns) was significantly higher at 100 µM ATP than at 1pM ATP application. The amplitude of the following peaks did not differ at 1pM ATP but were significantly lower at 100 µM ATP. Note that there was no difference between the mean amplitude of the first peak and the following peaks at 1pM ATP stimulation. Data are expressed as mean (SEM; N = 6). *p<0.05 was considered significant (unpaired t-test); § p<0.05 compared to respective control; n.s. = not significant.

We analysed the mean lag time of the first peaks after addition of ATP and found significant differences between high and low ATP concentrations (Fig. 5A). Also, the mean amplitude of the first peaks after high ATP application was significantly higher than after low ATP application (Fig. 5B). The mean amplitude of the first peaks was significantly higher than that of the following peaks at 10^−4^ mol/l ATP, while this was not the case at 10^−12^ mol/l ATP.

### Analysis of purinergic receptors involved

#### Agonist stimulation

**Figure 6:**
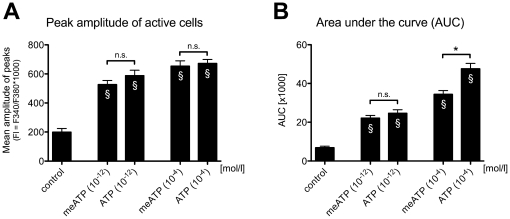
Agonist (α,β-methylene ATP; meATP) induced intracellular calcium response in sMF. (**A**) Mean amplitude of sMF evoked by α,β-methylene ATP at 1pm and 100 µM was significantly increased compare to control experiment, while there is no significant difference compared to ATP induced effect. Data are expressed as mean (SEM; N = 2). *p<0.05 was considered significant (unpaired t-test); § p<0.05 compared to respective control; n.s. = not significant. (**B**) The differences between 10^−4^ mol/l and 10^−12^ mol/l were significant for ATP and meATP (p>0.0001, t Test with Welch's correction for unequal variances).

ATP-induced calcium response was mimicked by α,β-methylene ATP (α,β-meATP), a P2X1 and P2X3 receptors agonist, at both used concentrations, 1pM (10^−12^ mol/l) and 100 µM (10^−4^ mol/l). While peak amplitudes were not different between α,β-meATP and ATP at the same concentrations (Fig. 6A), area under the curve (AUC), describing the sum of elevated calcium over time, was significantly different higher at 100 µM ATP stimulation (Fig. 6B).

#### (ii) Signal inhibition by specific antagonists

**Figure 7:**
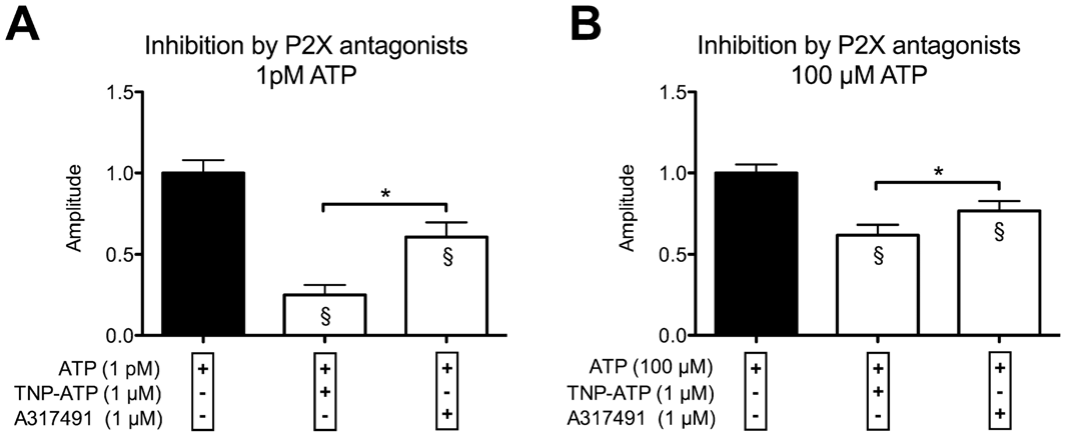
Inhibition of ATP induced intracellular calcium signals by purinergic receptor antagonists. (**A**) Normalized amplitude of active cells at 1pM ATP stimulation in the presence and absence of purinergic antagonists. (**B**) Normalized amplitude of active cells at 100 µM ATP stimulation in the presence and absence of purinergic antagonists. Data expressed as mean (SEM; N = 3). Amplitudes were normalized (see text). These antagonists inhibit the ATP-induced purinergic calcium signals by a competitive mechanism. We seperately incubated the cells for 20 minutes with the antagonists before the start of the recordings. In addition, the cells were rinsed with antagonist containing Ringer solution. *p<0.05 was considered significant (One-way ANOVA, Tukey's multiple comparison test); § p<0.05 compared to respective control; n.s. = not significant.

We used two different purinergic antagonists to investigate the contribution of P2X receptor subtypes to the response of the sMF. TNP-ATP (1 µM) selectively inhibits P2X1, P2X3 and P2X2/3 receptors and A-317491 (1 µM) selectively inhibits P2X3 and P2X2/3 receptors. ATP evoked calcium transients were partially attenuated by both purinergic antagonists. Antagonists alone showed no effect (tested in Part A of the experiments, see Fig. 2). Data were normalized to agonist stimulation, 10^−12^ mol/l (1pM) and 10^−4^ mol/l (10 µM) respectively (Fig. 7). The mean amplitude of calcium peaks was significantly reduced at both ATP concentrations. However, at 1pM ATP stimulation, TNP-ATP (74.98%) showed a significant higher inhibition than A-317491 (39.29%) (Fig. 7A), while at 100 µM ATP stimulation the inhibition by TNP-ATP and A-317491 was reduced by 38.1% and 23.4%, respectively (Fig. 7B).

#### (iii) Confocal immunofluorescence of purinergic receptors

**Figure 8:**
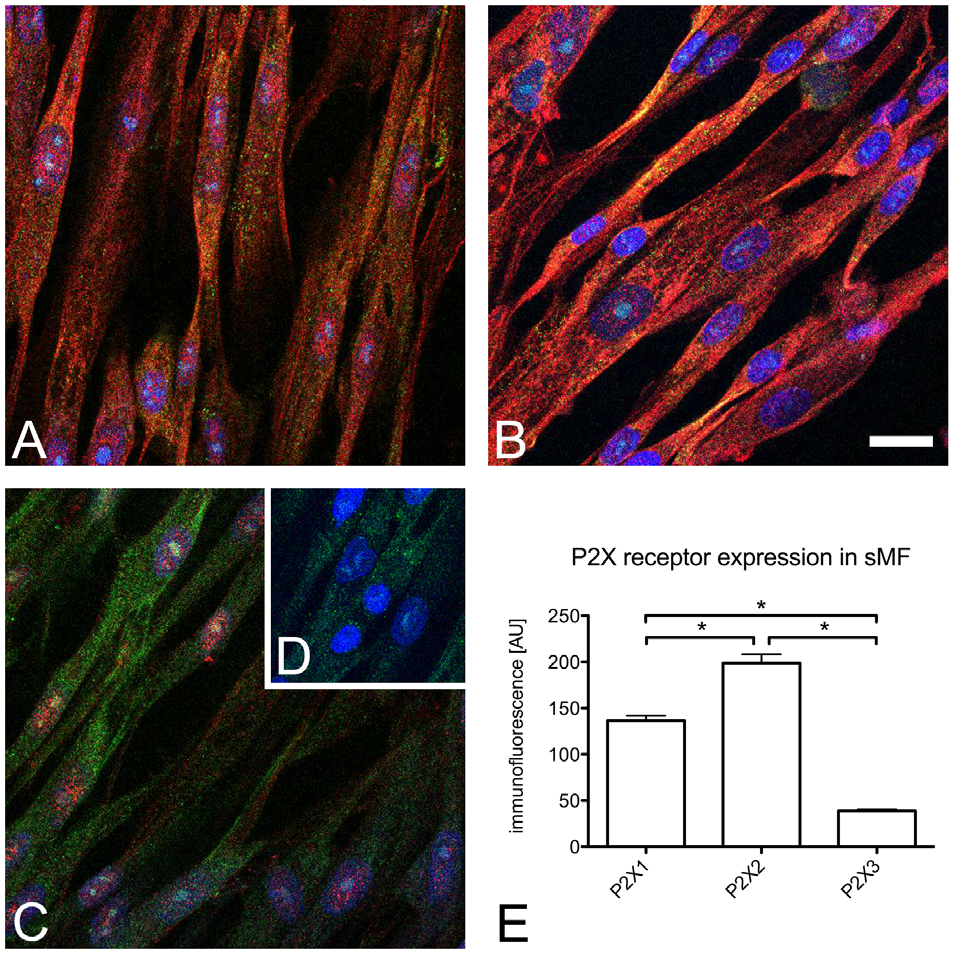
Confocal immunofluorescence of purinergic receptors (red) and alpha smooth muscle cell actin (aSMCA; green) in cultured sMF. (**A**) P2X1, (**B**) P2X2, (**C**) P2X3 labeling, (**D**) staining control, receptor specific antibody omitted. (**E**) Quantification of purinergic receptor immunoreactivity using confocal laser scanning microscopy. Scale bar in **B** indicates 20 µm and applies to **A-D**. Data expressed as mean (SEM; n = 105 cells, N = 3 exp.); *p<0.05 was considered significant (One-way ANOVA, Tukey's multiple comparison test).

The expression of purinergic receptor subtypes in sMF was examined by confocal immunofluorescence (Fig. 8A–E). Strong P2X1 (Fig. 8A) and P2X2 (Fig. 8B) receptor immunoreactivity (IR) was seen in cultured suburothelial myofibroblasts of human bladder, while P2X3 receptor-IR was significantly lower (Fig. 8C, D). The expression pattern of P2X receptors thereby was the same as measured in sMF in tissue slices of human bladders (Fig. S3).

## Discussion

Two levels of spontaneous activity in the bladder have to be considered; (i) spontaneous detrusor contractile activity, which is regularly observed in bladder strip organ bath preparations [18], [19] as well as in whole bladders [5], [6]; (ii) calcium activity within the lamina propria which can evoke detrusor contractions in the rat bladder and therefore suggest pacemaker activity of lamina propria myofibroblasts [13]. Alterations of spontaneous contractile activity of the bladder have been described in bladder strips from patients with idiopathic detrusor instability [19] and from patients with neurogenic detrusor overactivity [20]. In the latter, spontaneous contractile activity was not altered by removing the urothelium/suburothelium from the bladder strips which indicates that the spontaneous detrusor contractions were initialized within the detrusor itself [20]. In contrast, removal of the mucosa and submucosa inhibited spontaneous detrusor contractions in guinea pigs [18] and pigs [21] indicating a different underlying mechanism of generation.

In our experiments we found spontaneous calcium activity in 55.18%±1.65 of the cultured human sMF. This is slightly more than reported recently in cells freshly isolated from stable detrusor where 40% of the cells showed spontaneous calcium transients, but clearly less than in cells from overactive bladders (74%) [22]. It is especially interesting that the suburothelial myofibroblasts retain a constant level of spontaneous calcium activity even after several cell passages in culture.

To the best of the authors knowledge, there is no information on the number of spontaneous activity in human myofibroblasts. However, Wu et al. [14] found spontaneous inward currents, preceded by spontaneous Ca^2+^ rises, in 45% of freshly isolated guinea pig sMF, which is in good agreement with our results in the long term cultured human sMF.

### The role of ATP in bladder afferent signaling

Several reports have documented the use of micromolar concentrations of ATP to elicit maximal responses of Ca^2+^ activity. Having referred to literature [23], we used 100 µM ATP in our study as maximum ATP concentration. The measurements above 10^−8^ mol/l are consistent with the results described in the literature [24], [25], where the lowest ATP concentration that evoked calcium transients was reported to be 10^−8^ M level with an EC50 between 1 and 10 µM.

ATP is released from urothelium during bladder filling and this signal is detected by sensory nerves and sMF. Two studies reported that ATP concentration released from urothelium was at very low level (pmol/l) during the initial filling phase in rat bladder and cultured urothelial cells [2], [4]. Here we show for the first time, that even sub-picomolar concentrations of extracellular ATP are effective in modulation of the suburothelial myofibroblast activity; this suggests a possible functional connection between urothelium and sMF.

Interestingly, in our study the profile, calcium transients in sMF display a different pattern at low and high ATP concentration (Figs. 4 and 5). The calcium transients could be evoked rapidly with a short lag time by high ATP concentration. This ATP induced effect was also observed on other cell types [26]. Though those directly evoked calcium transients were absent at low ATP concentrations, there was still a significant increase in peak frequency and amplitude. This means that even pico-molar concentrations of ATP, as expected during initial phase of bladder filling, can modulate the pattern of the spontaneous activity in sMF. We conclude, that sMF could function as very sensitive detectors for mechanical stress in the bladder wall. Since we used almost confluent cell cultures the low ATP-effect may be a function of the whole syncytium of sMF. Further studies on single sMF or using gap junction inhibitors are needed to clearify this point.

ATP exerts its effects via two general classes of purinergic receptors, P2X and P2Y [27]. Here we report the expression of P2X (P2X1, P2X2, P2X3) receptors in cultured human sMF, which is in agreement with our previous study [16] and a recent study demonstrating P2X3 receptor expression in suburothelial myofibroblasts from normal human bladder [28]. Our previous study also showed that carbachol and ATP-evoked intracellular calcium transients depend on extracellular calcium, implying that P2X receptors are involved in ATP-induced intracellular calcium transients [16]. The bi-phasic course of the dose-response curve could be explained with distinct purinergic receptors involved.

In the present study the ATP induced calcium transients of sMF were mimicked by α,β-methylene ATP (P2X1, P2X3 selective agonist).

For initial analysis we used two selective purinergic antagonists to investigate the contribution of P2X receptor subtypes to the response of the sMF. TNP-ATP used at 1 µM selectively inhibits P2X1, P2X3 and P2X2/3 receptors, while A-317491 at 1 µM selectively inhibits P2X3 receptors with negligible effect on P2X1 and P2X2 receptors [29], [30]. We used antagonist concentrations, which were 10–100 fold higher than the IC50 reported in literature to ensure sufficient and reproducible inhibition [29]. Calcium transients were only partially attenuated by all antagonists, at both low and high ATP concentrations.

At high ATP concentration (100 µM) we found about 20% inhibition of the signal by A-317491 and about 40% inhibition by TNP-ATP (Fig. 7B). This indicates equal percentual contribution of P2X1 and P2X3 receptors.

Interestingly the effects of antagonists were stronger at low ATP concentration (1pM). A-317491 showed about 40% inhibition and TNP-ATP inhibited up to 75% of the ATP induced calcium amplitude (Fig. 7A).

Those experiments demonstrate that P2X1 and P2X3 receptors are involved in ATP signaling in sMF. However, a significant part of ATP signal seems to be a significant part of ATP signal mediated by other purinergic receptors. Due to lack of available P2X2 receptor antagonists it remains unclear, whether the P2X2 receptor is involved in ATP signaling as suggested by the high expression found in immunocytochemical examination (Fig. 8B, D).

Our findings dissent from the results of other groups providing evidence to that only metabotropic P2Y receptors, especially P2Y6 is involved in ATP mediated calcium signalling in isolated suburothelial myofibroblasts of the guinea pig [14], [31]. In immunohistochemical studies of purinergic receptor expression in sMF in the human bladder we found a corresponding expression pattern (P2X2>P2X1>P2X3; Fig. S3) but very little P2Y expression (data not shown) supporting the notion that the cutured cells are comparable to human sMF as found *in vivo*. The cause for the low P2Y receptor expression has to be elucidated in further studies.

In the present study we exclusively examined the ATP effects on cultured sMF. Whether the high ATP sensitivity found *in vitro* is also present *in vivo* remains to be investigated. However, our findings do support the notion that sMF have the potential to act as very sensitive ATP sensors.

### Conclusions

Human cultured suburothelial myofibroblasts show spontaneous calcium activity and we demonstrate, for the first time, that ATP can modulate this spontaneous activity at very low ATP concentrations involving P2X receptors. These findings support the notion that sMF are competent, and very sensitive, to register bladder fullness, which predestines them for the modulation of the afferent bladder signaling in normal and pathological conditions.

## Supporting Information

Figure S1 **Distribution and immunocytochemistry of suburothelial myofibroblasts.** Human unaltered bladder paraffin sections were incubated over night with monoclonal mouse antibodies: anti-aSMCA (IgG2a, 1∶2000; Sigma-Aldrich), anti-vimentin (1∶100; Sigma-Aldrich) or anti-CD117 (c-Kit, 1∶100; DAKO, Glostrup, Denmark) and visualized with Envision-Kit™ (DAKO) using AEC substrate chromogen (3-Amino-9-ethylcarbazole in 2.5% N,N-dimethylformamide; red color). Nuclei were stained with Mayer's hematoxylin. (**A**) Numerous aSMCA positive cells are present in the lamina propria directly underneath the urothelium. The staining pattern resembles that of vimentin (**B**). CD117 immunoreactivity was confined to only few suburothelial elongated cells (**C, arrowheads**) and to cells in-between smooth muscle cell bundles (**D, arrows**). Urothelial cells regularly showed light CD117 immunoreactivity with only few more intensely stained elongated cells within the urothelial cell layer (**arrow in C**). (**E**) Staining control showed no AEC background staining. U – Urothelium; the scale bar in **D** indicates 100 µm and applies to **A-E**.
(TIF)

Figure S2 **Cell culture morphology.** Phase contrast micrographs. (**A**) Typical suburothelial myofibroblast culture; note the characteristic morphology of sMF in this sub-confluent cell culture; (**B**) urothelial cell culture demonstrating typical cobblestone-like morphology of human urothelial cells. The scale bar in **B** indicates 100 µm and applies to **A and B**.
(TIF)

Figure S3 **P2X receptor expression.** Confocal immunofluorescence analysis of tissue sections of control bladders. Data expressed as mean (SEM; n = 105 cells, N = 3 patients); *p<0.05 was considered significant (One-way ANOVA, Tukey's multiple comparison test).
(TIF)
