# Supplementary figures and images for: ATP Enhances Spontaneous Calcium Activity in Cultured Suburothelial Myofibroblasts of the Human Bladder
Source: PLoS One. 2011 Oct 5;6(10):e25769. doi: 10.1371/journal.pone.0025769 (PMC3187810; doi:10.1371/journal.pone.0025769)

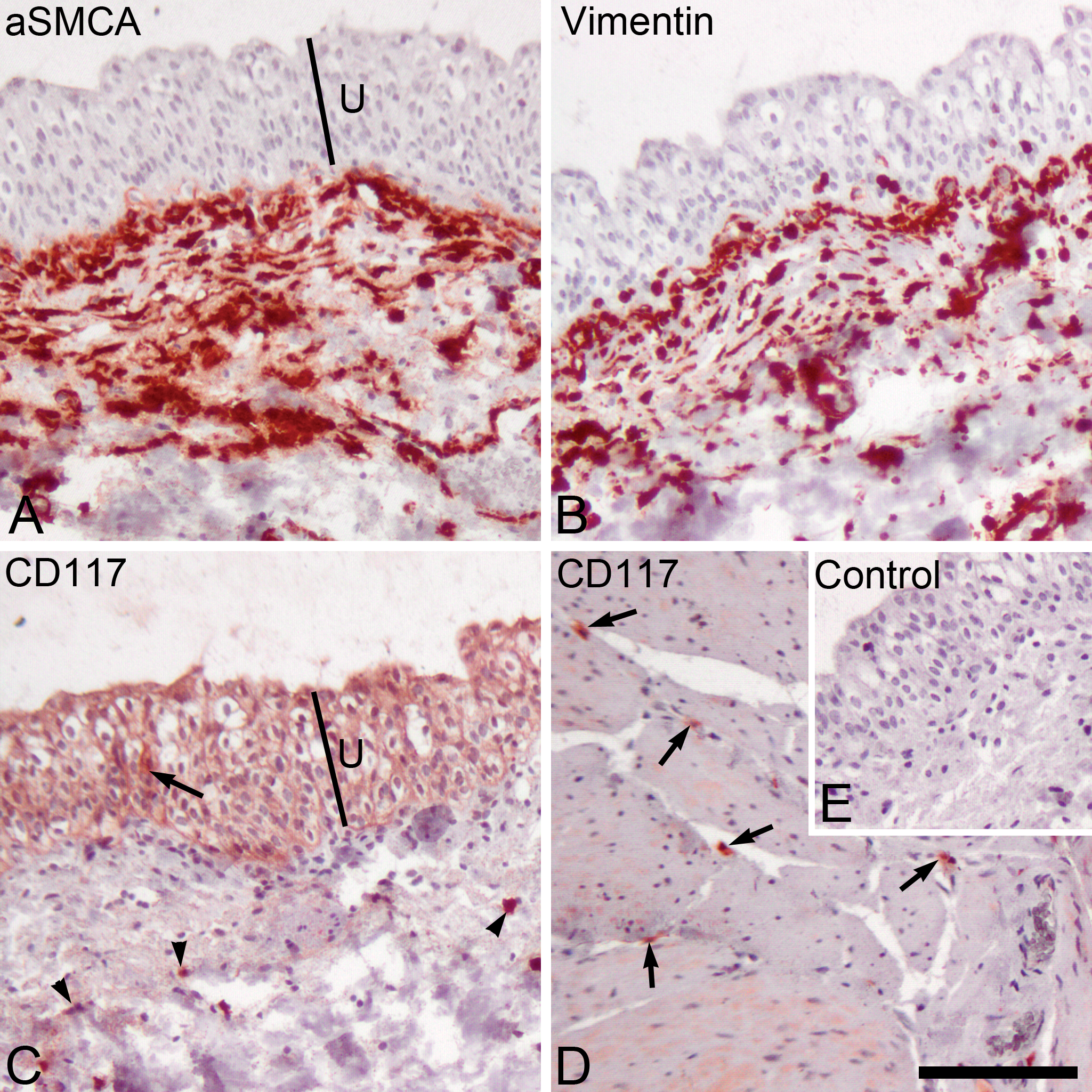

Supplement: Figure S1 — Distribution and immunocytochemistry of suburothelial myofibroblasts. Human unaltered bladder paraffin sections were incubated over night with monoclonal mouse antibodies: anti-aSMCA (IgG2a, 1∶2000; Sigma-Aldrich), anti-vimentin (1∶100; Sigma-Aldrich) or anti-CD117 (c-Kit, 1∶100; DAKO, Glostrup, Denmark) and visualized with Envision-Kit™ (DAKO) using AEC substrate chromogen (3-Amino-9-ethylcarbazole in 2.5% N,N-dimethylformamide; red color). Nuclei were stained with Mayer's hematoxylin. (A) Numerous aSMCA positive cells are present in the lamina propria directly underneath the urothelium. The staining pattern resembles that of vimentin (B). CD117 immunoreactivity was confined to only few suburothelial elongated cells (C, arrowheads) and to cells in-between smooth muscle cell bundles (D, arrows). Urothelial cells regularly showed light CD117 immunoreactivity with only few more intensely stained elongated cells within the urothelial cell layer (arrow in C). (E) Staining control showed no AEC background staining. U – Urothelium; the scale bar in D indicates 100 µm and applies to A-E. (TIF) [file pone.0025769.s001.tif]

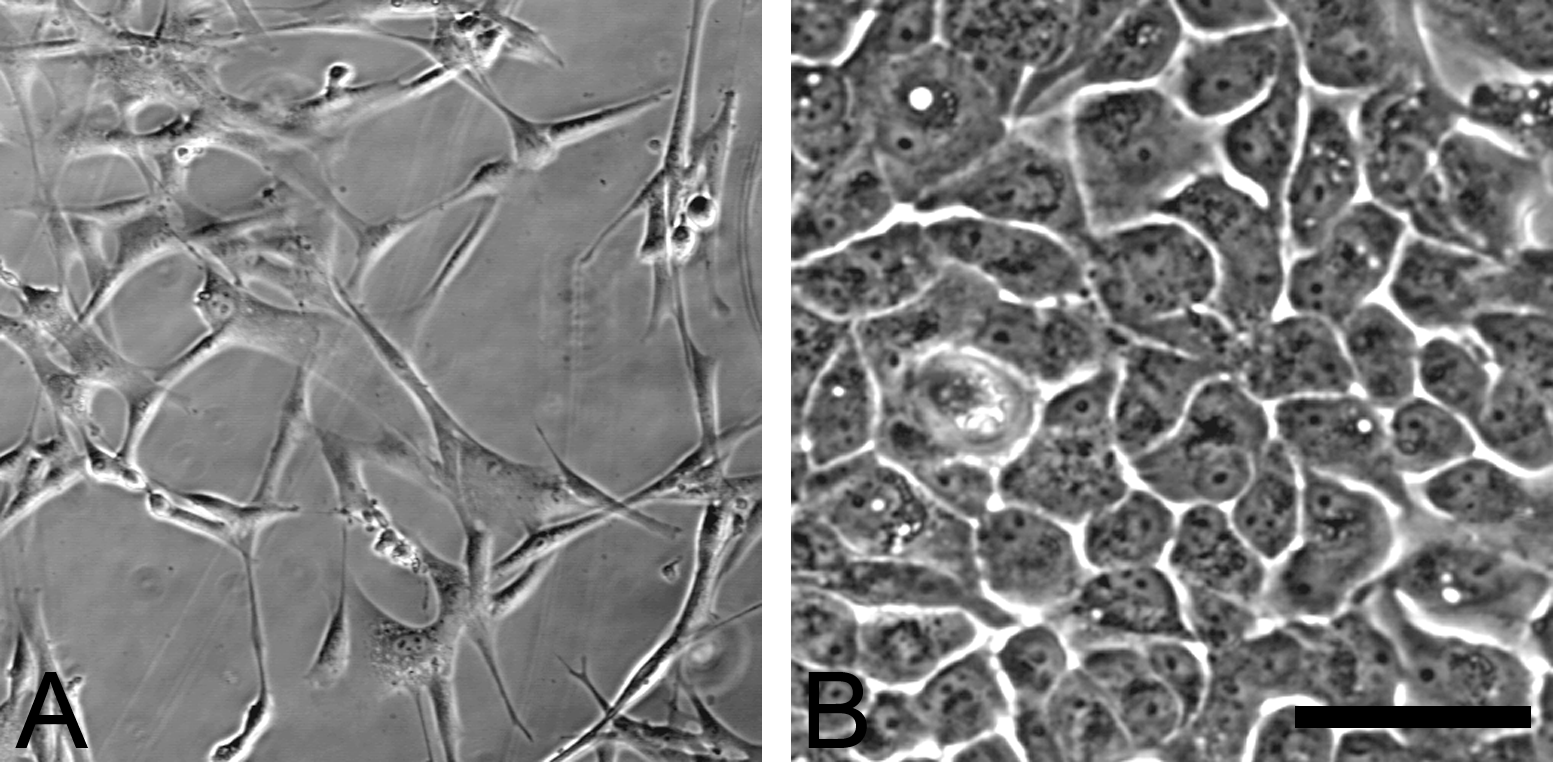

Supplement: Figure S2 — Cell culture morphology. Phase contrast micrographs. (A) Typical suburothelial myofibroblast culture; note the characteristic morphology of sMF in this sub-confluent cell culture; (B) urothelial cell culture demonstrating typical cobblestone-like morphology of human urothelial cells. The scale bar in B indicates 100 µm and applies to A and B. (TIF) [file pone.0025769.s002.tif]

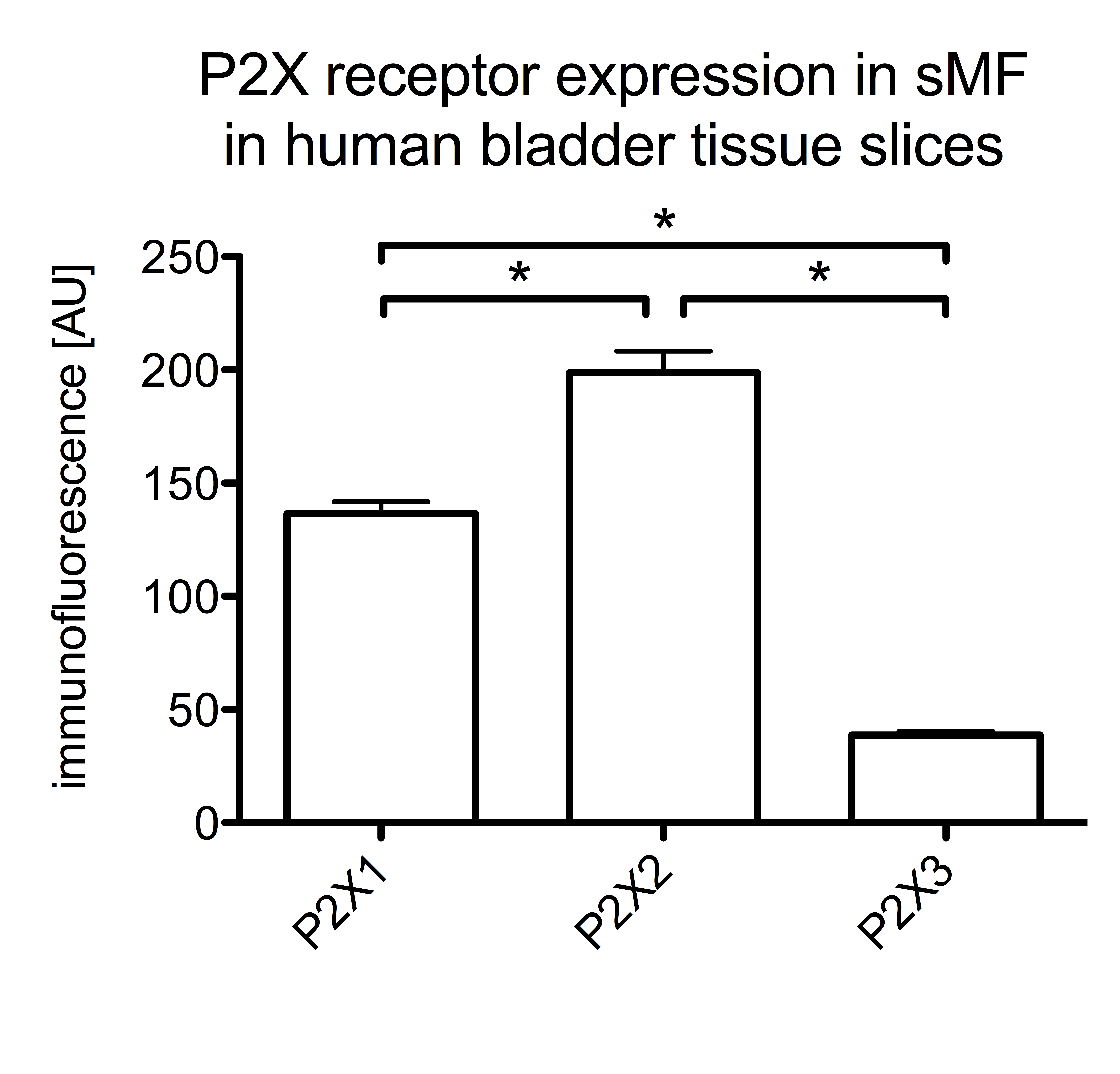

Supplement: Figure S3 — P2X receptor expression. Confocal immunofluorescence analysis of tissue sections of control bladders. Data expressed as mean (SEM; n = 105 cells, N = 3 patients); *p<0.05 was considered significant (One-way ANOVA, Tukey's multiple comparison test). (TIF) [file pone.0025769.s003.tif]
